# Evaluation of polyester high-tenacity fabric and carbon nanotube reinforcements for improving flexural response in concrete beams

**DOI:** 10.1038/s41598-024-76729-8

**Published:** 2024-11-06

**Authors:** M. S. El-Feky, Amr H. Badawy, Khaled Mohamed Seddik, Sarah Yahia

**Affiliations:** 1 Civil Engineering Department, Engineering & Renewable Energy Research Institute, National Research Centre, Giza, Egypt; 2 Clothing and Knitting Industrial Research Department, Textile Research and Technology Institute, National Research Centre, Giza, Egypt; 3 Department of Spinning and Weaving Engineering, Textile Research and Technology Institute, National Research Centre, Giza, Egypt

**Keywords:** Textile reinforcement concrete (TRC), Carbon nano tube, Polyester high, Tenacity, Weave structures, Engineering, Materials science, Nanoscience and technology

## Abstract

This research scrutinized the effectiveness of utilizing polyester high tenacity fabrics to enhance the functionality of concrete panels. Two distinct woven fabrics with comparable strength resistance were fabricated and assessed. Concrete beams were compared in their original form and those reinforced with woven fabrics, along with beams reinforced with carbon nanotubes (CNTs) (B, BC2, BC4, BS1, and BS2). Results indicated that the textile-reinforced concrete panels displayed notably greater energy absorption capabilities post-failure under flexural loads in comparison to the control and CNT-reinforced panels. This enhanced performance was credited to the development of multiple cracking patterns in the textile-reinforced panels. The flexural behavior of the textile-reinforced panels was characterized by four discernible phases: a linearly increasing segment, a crack propagation phase featuring multiple cracking, a post-cracking phase with reduced stiffness, and ultimately, failure due to fabric rupture or debonding. Conversely, the control and CNT-reinforced panels exhibited a more brittle response post-initial cracking, with a limited number of cracks and reduced deformation capacity. The performance of the textile samples was largely unaffected by their specific characteristics, except for the fabric wrapping angle. The introduction of 0.04% CNTs marginally enhanced crack flexural resistance compared to the control and 0.02% CNT panels, owing to the varied distribution of CNTs within the matrix. Overall, the textile-reinforced concrete panels demonstrated superior load-bearing capacity, ductility, and energy absorption when compared to the other reinforcement techniques examined.

## Introduction

Reinforced concrete (RC) is widely utilized in construction as a primary material. However, various factors such as inadequate design, heavy loads, and harsh environmental conditions can lead to diminished load-bearing capacity in RC structures, necessitating the implementation of strengthening techniques. Strengthening is crucial to address challenges associated with occupant characteristics, material degradation, and the need for enhanced safety measures^1,2^. Beam elements, commonly subjected to sustained loads during their service life, often exhibit low flexural performance and may require strengthening. To ensure ductility and resistance to bending under sustained loads, Reinforced Concrete Beams (RCBs) are typically designed as ductile members. Ductile failure is generally preferred over brittle failure for RCBs, and increasing the reinforcement ratio in the design can improve their flexural performance and ductility^3^.

The integration of carbon nanotubes (CNTs) into the concrete matrix holds promise for further enhancing the flexural response of RC elements. CNTs possess exceptional mechanical, thermal, and electrical properties, making them attractive as micro-scale reinforcements. Previous studies have examined the flexural strength of cement mortars incorporating multi-walled carbon nanotubes (MWCNTs) and have reported significant improvements in flexural strength and stress-intensity factor of the cement composites. For instance, the addition of 0.1% CNTs by weight of cement resulted in a 7% increase in flexural strength and a 72% increase in flexural modulus compared to CNT-free cement mortars. Other researchers investigated the effect of MWCNTs on the mechanical properties of cement mortars, observing improvements of 87% in flexural strength, 95% in Young’s modulus, and 77% in fracture toughness. However, the addition of CNTs to cement composites presents challenges such as agglomeration of nanotubes, weak cohesion between CNTs and the cement matrix, and the high cost of CNTs, which limits their widespread use in cement composites^4–8^.

Technical textiles have gained increasing prominence in recent years, particularly in the field of structural engineering. These specialized textiles are being widely adopted as innovative reinforcements for concrete structures, representing a promising alternative to traditional steel reinforcement. Textile-reinforced concrete (TRC) considers as one of the external bonded reinforcement technique refers to the bonding with fabrics that offers several advantages over conventional reinforced concrete, including reduced weight, improved corrosion resistance, and enhanced design flexibility^9,10^.

The key factors in the performance of TRC are the choice of the yarn types and weave structures that allow this fabric to fulfil specific requirements that amplify its mechanical properties such as strength, solidity, and longevity, aiming to mitigate this challenge and augment its tensile strength. Therefore textile-reinforced concrete (TRC) is preferred over FRP sheets, or steel plates, which have many weakness properties such as difficulty in shape, weight, handle and transport, they are about 6-meter length, which requires joints.

Diversified fiber types such as glass, carbon, aramid, and polypropylene, are now essential to modern concrete constructions because they allow the buildings to withstand significant stress and strain. Furthermore, the use of various fiber materials, including as glass and carbon fibers, which provide outstanding resistance to corrosion and a fantastic strength-to-weight ratio, is becoming more and more popular in the concrete construction industry^11,12^.

Synthetic fibers, particularly polyester, are the most widely used due to their superior mechanical properties and consistent behavior compared to natural fibers^11^. Polyester fibers have been found to enhance the mechanical properties of concrete and other applications, such as toughness, tensile strength, flexural strength, and resistance to shrinkage cracking^14^ and Ref.^15^. Thus, polyester high-tenacity fabrics have emerged as a viable option, exhibiting desirable mechanical properties such as high tensile strength and low elongation^14–16^.

Weave structures of the textile-reinforced concrete (TRC) play an important role in offering benefits like resistance to rust, lightweight, and the ability to be shaped and applied close to the surface for the reinforcement concrete^12–17^. Weave structures imply the interlacements among warp (vertical) and weft (horizontal) yarns that effect intensively on the multiple woven fabric characteristics; i.e. areal density, thickness, force resistance strength, stretch-ability, air and water permeability, etc. The classifications of weave structures are based on the interlacing pattern e.g. plain structure, twill structure, satin structure, and others that determine the fabrics’ performance^18^, and Ref.^19,20^.

This research aims to conduct a comparative assessment of the flexural behavior of concrete beams reinforced with polyester high-tenacity fabrics and CNT-based composites. The findings will contribute to a better understanding of the optimal textile reinforcement configuration and the synergistic effects of combining polyester high-tenacity fabrics and CNT reinforcements for enhancing the flexural response of concrete structures.

## Experimental work

### Textile properties and preparation techniques

To fulfill the purpose of the investigation, two alternative high tenacity polyester multifilament yarns were tested as shown in Table 1. Based on the results of yarn characteristics, two distinct fabrics with approximately the same force resistance were manufactured. Plain and twill weaves are the basic structures of the produced fabrics. Picanol (Rapier) loom machine equipped with STAUBLI dobby device was utilized. The loom specifications are associated with 190 cm working width, one warp beam, one cloth beam, and 600 rpm (maximum speed). Table 2 elucidates the scheme of produced fabrics.

**Table 1:** The characteristics of utilized polyester yarns.

Characteristics	Polyester high-tenacity yarns	
	BS1	BS2
Yarn count [tex]	250	500
Max. tencity. [Newton/tex]	0.3669	0.380167
Max. strain. [%]	9.863333	6.003
Max. load [Kilo Newton]	0.098367	0.1832

**Table 2:** The scheme of produced fabrics.

Samples	Warp count	Weft count	Density		Structure	Warp cover factor*	Weft cover factor*	Cloth cover factor*
			Warp/cm	Weft/cm				
Plain sample BS1	500 Tex*	250 Tex*	6	4		13.41	6.32	19.73
Twill sample BS2	250 Tex*	250 Tex*	9	9		14.23	14.23	28.46

*Tex: a metric system used to measure the linear mass density of a yarn. It denotes the weight in grams of 1000 m of yarn.

*Cover factor: specifies the amount to which the area of a fabric is covered by a single set of yarns (warp or weft), and calculated by the formula:

*Cloth cover factor: identify the total cover factor for the woven fabric by adding the warp cover factor to the weft cover factor.

#### Laboratory tests

Due to identify the characteristics of produced samples, several testing were occurred as follows:

(A) Structural characteristics: include measurements of fabric weight (Areal density) with accordance to ASTM D3776 utilizing an electronic balance with an accuracy of four digits, and fabric thickness with accordance to ASTM D1777 associated with portable thickness gauge^20^ and Ref.^21^.
(B) Utility characteristics: include measurements of Air permeability was executed utilizing Toyoseiki (JIKA) instrument, with accordance to ASTM D737, and Water permeability with accordance to ASTM D4491-99a by using water permeability tester SDL ATLAS^22^ and Ref.^23^.
(C) Mechanical characteristics: include measurements of tensile and elongation with accordance to ASTM D5034 using Instron tensile testing instrument (CRE) model 3345^24^.

Before testing all samples were conditioned with accordance to standard test method ISO 139 for testing textile, as the samples placed in a relaxed state for 24 h at an ambient temperature of 20 ± 2 °C and a relative humidity of 65 ± 2%^25^.

For each sample, three replicates were occurred for every test. The results were collected, tabulated, and the average was calculated as well.

### Analytical tools

Due to present the characteristics of produced fabrics, all results were exhibited using a chart graph. The significant/insignificant effect of parameters was analyzed statistically according to p-value ≤ 0.05. Furthermore, the radar chart area of each fabric was calculated and plotted in order to determine the performance of proposed fabric.

### Concrete preparation & methodology

#### Methodology

The methodology employed in this research involved an experimental program conducted on simply supported concrete beams. The beams were categorized into different groups based on the presence or absence of carbon nanotubes (CNTs) and external strengthening with two types of textiles. The designations for the samples were B, BC2, BC4, BS1, and BS2, respectively.

All specimens underwent static vertical load testing at the laboratories of the National Research Centre (NRC) in Egypt. The mechanical properties of the materials used, as well as details regarding beam geometry, materials, casting, and testing procedures, are described in the subsequent section. The parameters studied included the ratio of CNTs (0%, 0.02%, and 0.04%) in the concrete beams and the external strengthening of simply supported concrete beams using the two textile types.

#### Description of test specimens

**Fig. 1:**
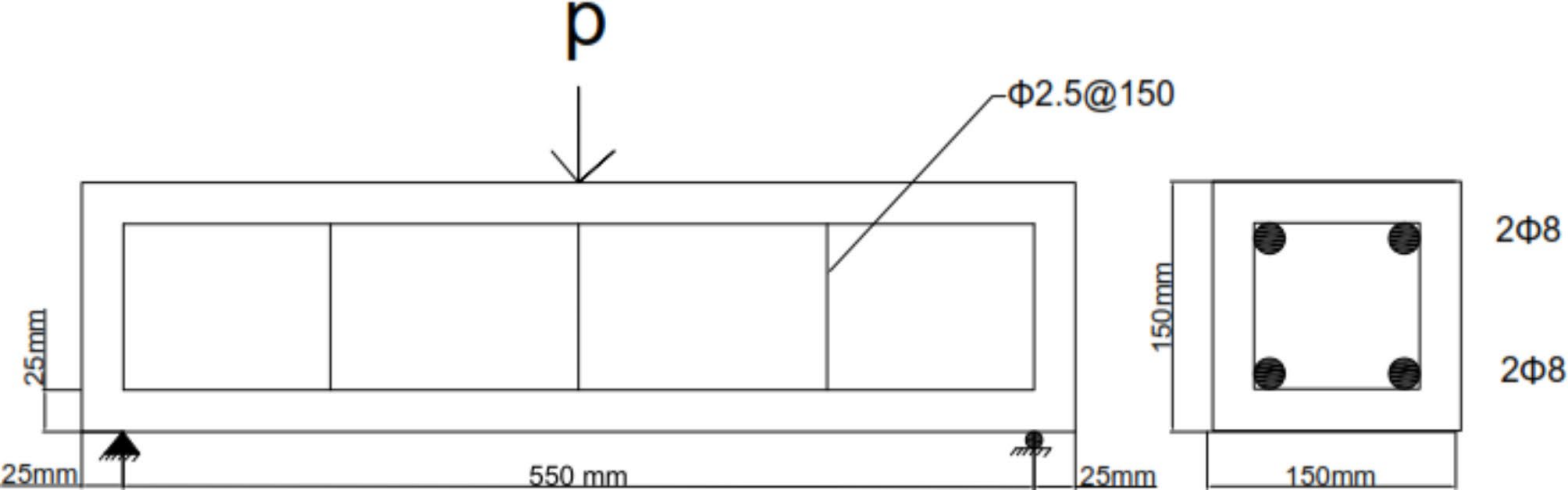
Reinforcement details of concrete beams with and without (CNT).

**Fig. 2:**
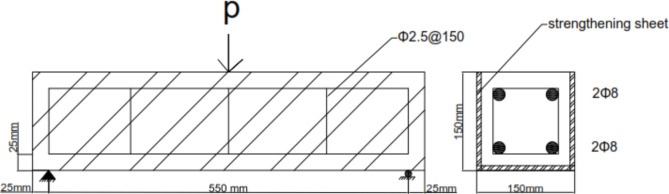
Reinforcement details of concrete simply supported beam strengthened with the two textile types.

The experimental work involved testing a total of four simply supported concrete beams subjected to flexural loading. These beams were divided into two groups for the experimental program. In the first group, the objective was to study the flexural performance of three concrete beams with 0.02% and 0.04% CNTs, respectively and a third beam as control beam consisted of concrete beams prepared using the same materials and mixing ratios as the reinforced beams, but without any additional reinforcement (textile or carbon nanotubes). In the second group, the focus was on evaluating the residual flexural performance of concrete beams externally strengthened with two textile types. All beams shared the same overall dimensions of 150 mm width, 150 mm depth, and 600 mm length, as well as identical longitudinal reinforcement and shear reinforcement. Stirrups, depicted in Fig. 1, consisting of 2.5 mm diameter bars placed at 150 mm intervals, were incorporated to prevent potential shear failure. The beams were simply supported with a clear span of 550 mm. For concrete beams with 0.02% and 0.04% CNT ratios, the CNTs were added to the concrete mixture during casting, while two 8 mm diameter bars served as the main reinforcement for the concrete beams with added mild steel, as shown in Fig. 2. The externally strengthened concrete simply supported beam was represented by the two textile types under investigation. The curing process was also identical for all groups in water tank for 28 days. Specific details of each specimen are provided in Table 3.

**Table 3:** Details of tested beams.

Beam	AS	AŚ	%CNT	Textile
B	2Φ8	2Φ8	0	–
BC2	2Φ8	2Φ8	0.02	–
BC4	2Φ8	2Φ8	0.04	–
BS1	2Φ8	2Φ8	0	T1
BS2	2Φ8	2Φ8	0	T2

The specimens designate were in the form (B, BC2, BC4 or BS). B, refers to the concrete beams, (C) refers to the carbon nano-tube, and (2 or 4) refers to the 0.02, and 0.04% (CNT) ratios respectively, and S refers to beam strengthened with the two textile types.

### Concrete properties and mix preparation

The reinforcing steel employed in the experimental work consisted of stirrups made of steel bars with a diameter of 2.5 mm, while the longitudinal reinforcement utilized deformed grade mild steel bars with a diameter of 8 mm.

In all the tested concrete beams, Portland cement of ASTM Type I was used. The concrete production involved a mixture of aggregates, with fines and coarse aggregates comprising 35% and 65% by volume, respectively. To enhance the workability of the concrete and facilitate the dispersion of carbon nanotubes (CNTs) within the concrete mix, a poly-carboxylate admixture (Glenium C315 SCC) was incorporated. Refer to Table 4 for further details.

**Table 4:** Concrete mixtures composition by weight (kg) per 1 m^3^.

Cement	Agg. coarse	Agg. fine	W	CNT	S.P	Fcu *N*/mm_2_
450	1168	629	180	0	3.3	40
450	1168	629	180	0.09	3.3	45
450	1168	629	180	0.18	3.3	50

### Beams testing setup

**Fig. 3:**
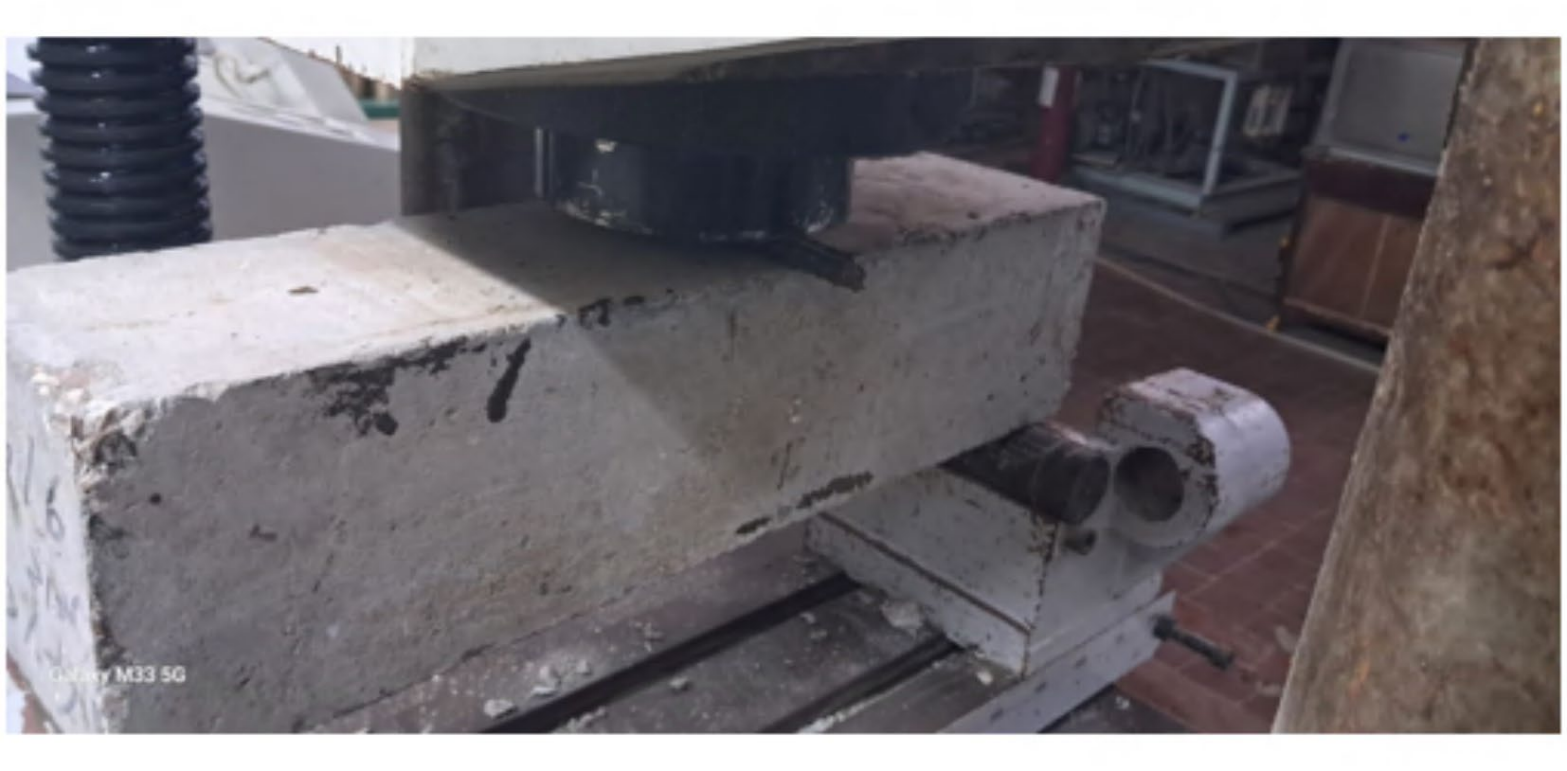
The instrumentation in the beam.

**Fig. 4:**
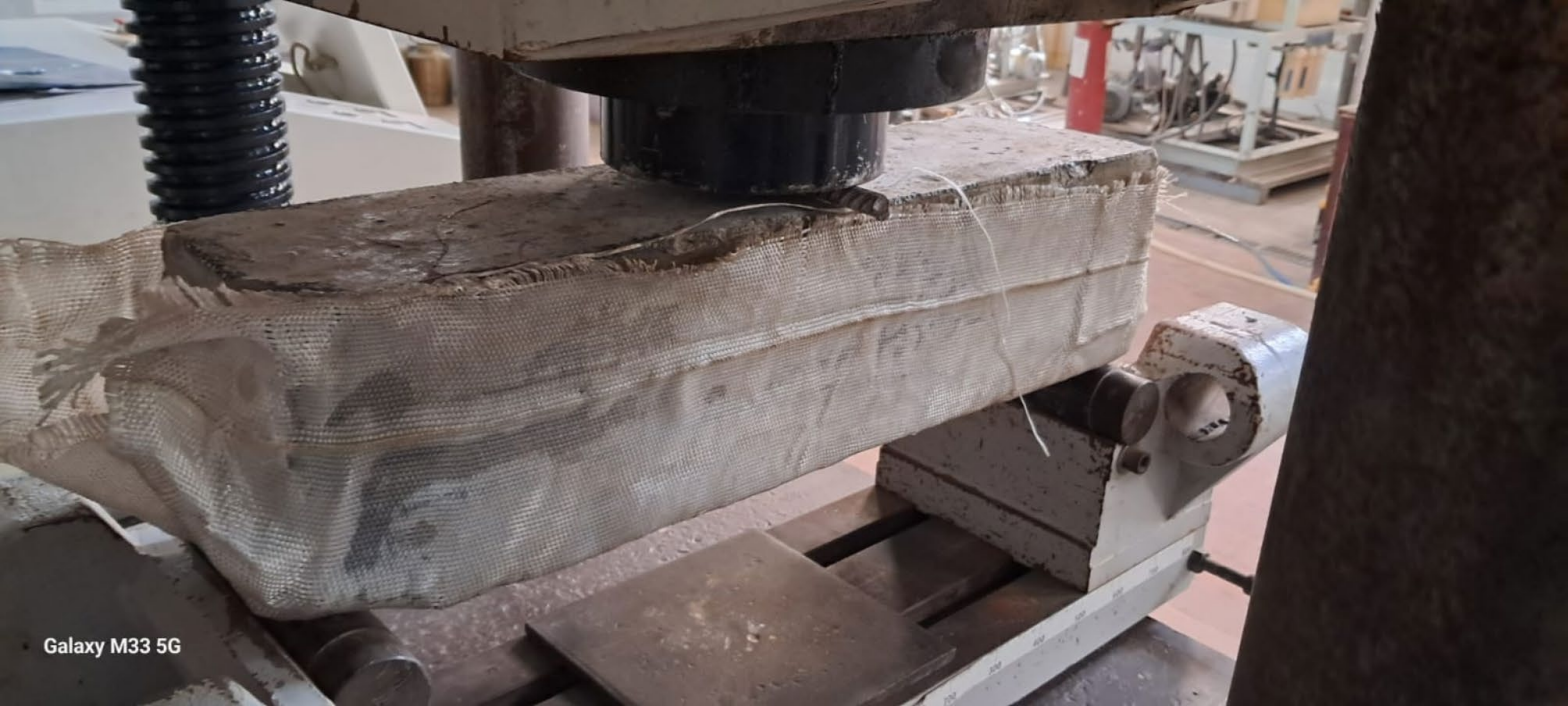
The concrete simply supported beam strengthened with type1 textile and glued by epoxy BS1.

**Fig. 5:**
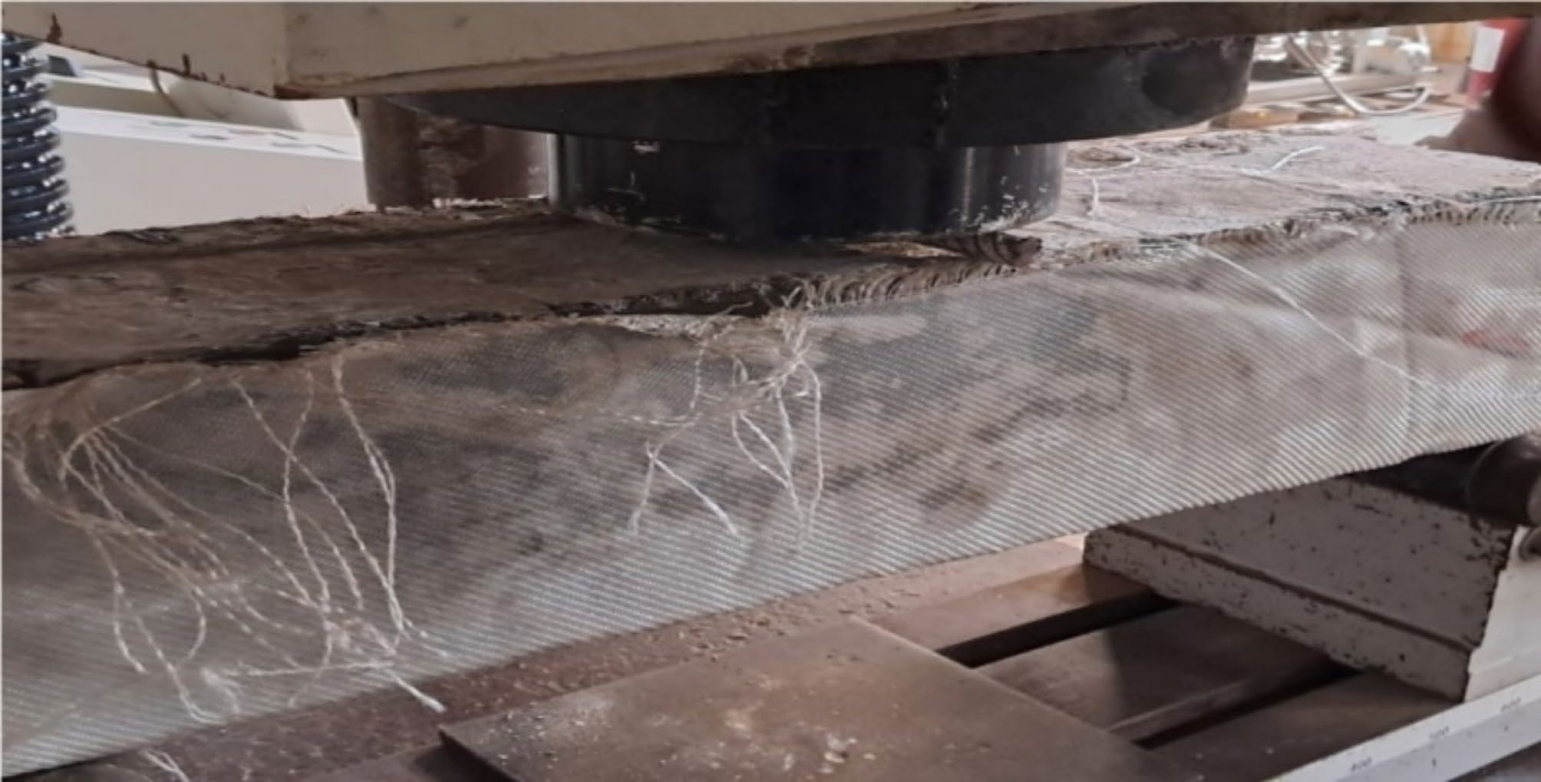
The concrete simply supported beam strengthened with type2 textile and glued by epoxy BS2.

To subject the beams to a point load at the mid-span, hydraulic jacks with a capacity of 1000-kN were employed. The specimens were supported by two steel rods, utilizing one free rod and one restrained rod to simulate a roller support and a hinged support, respectively. The beams were tested until failure, as illustrated in Fig. 3. Additionally, Figs. 4 and 5 depict the concrete simply supported beam externally strengthened with the two textile types.

## Results and discussions

### Textile structural characteristics

**Fig. 6:**
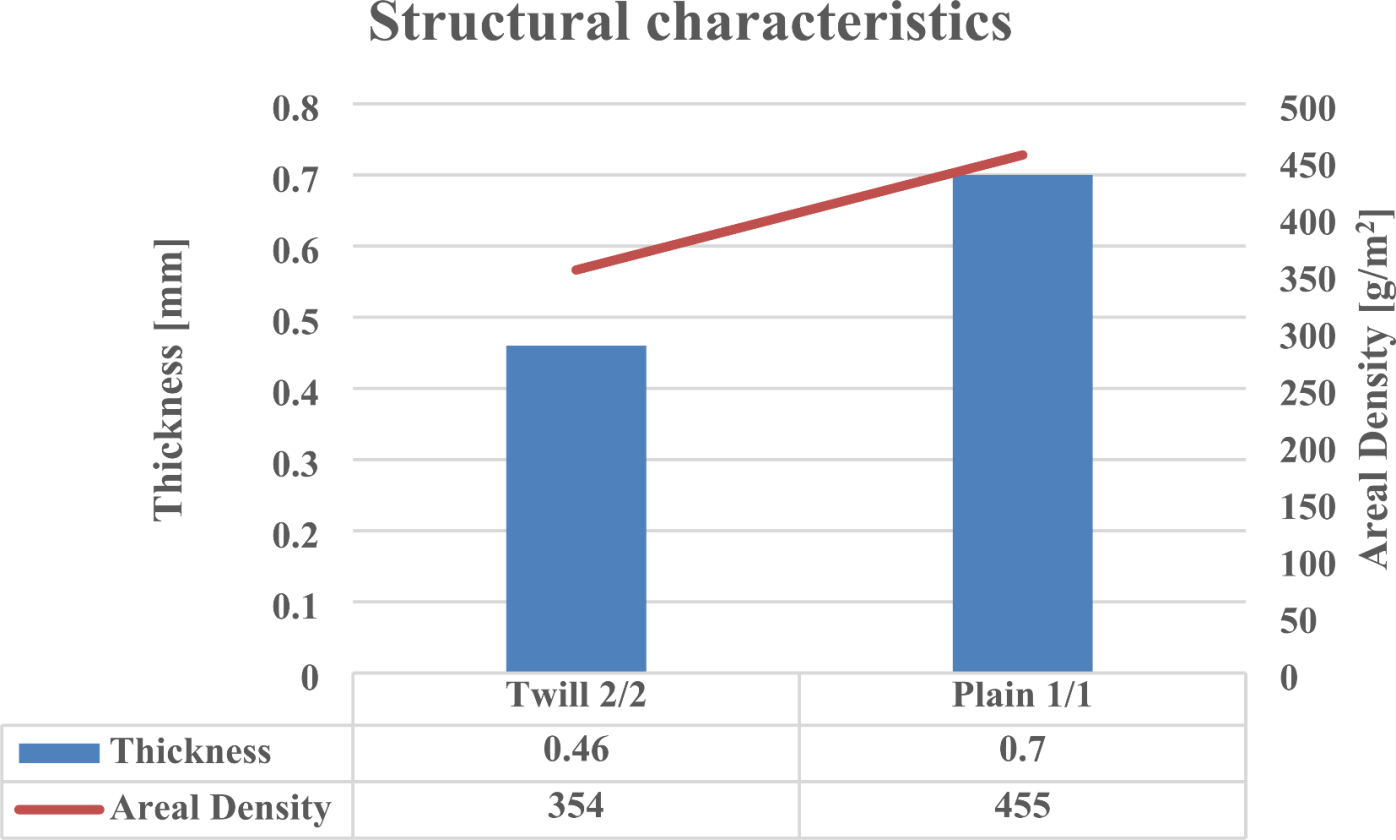
The structural characteristics of the produced woven fabrics.

The results demonstrate that although the plain woven structure composites with a lower yarns density (4 warp yarns/cm × 6 weft yarns/cm) cause in decreasing the cloth cover factor than the twill woven structure, it obtains a higher areal density and thickness. The interpretation could relate to the linear density of yarns, which increased by raising the count number of yarn in a direct system (Yarn count is measured as the weight of yarn per unit length, a higher value signifies a heavier yarn) as well as growing in yarn diameter that reflects on fabric weight and height. Figure 6 presents the structural characteristics of the produced woven fabrics.

### Utility characteristics

**Fig. 7:**
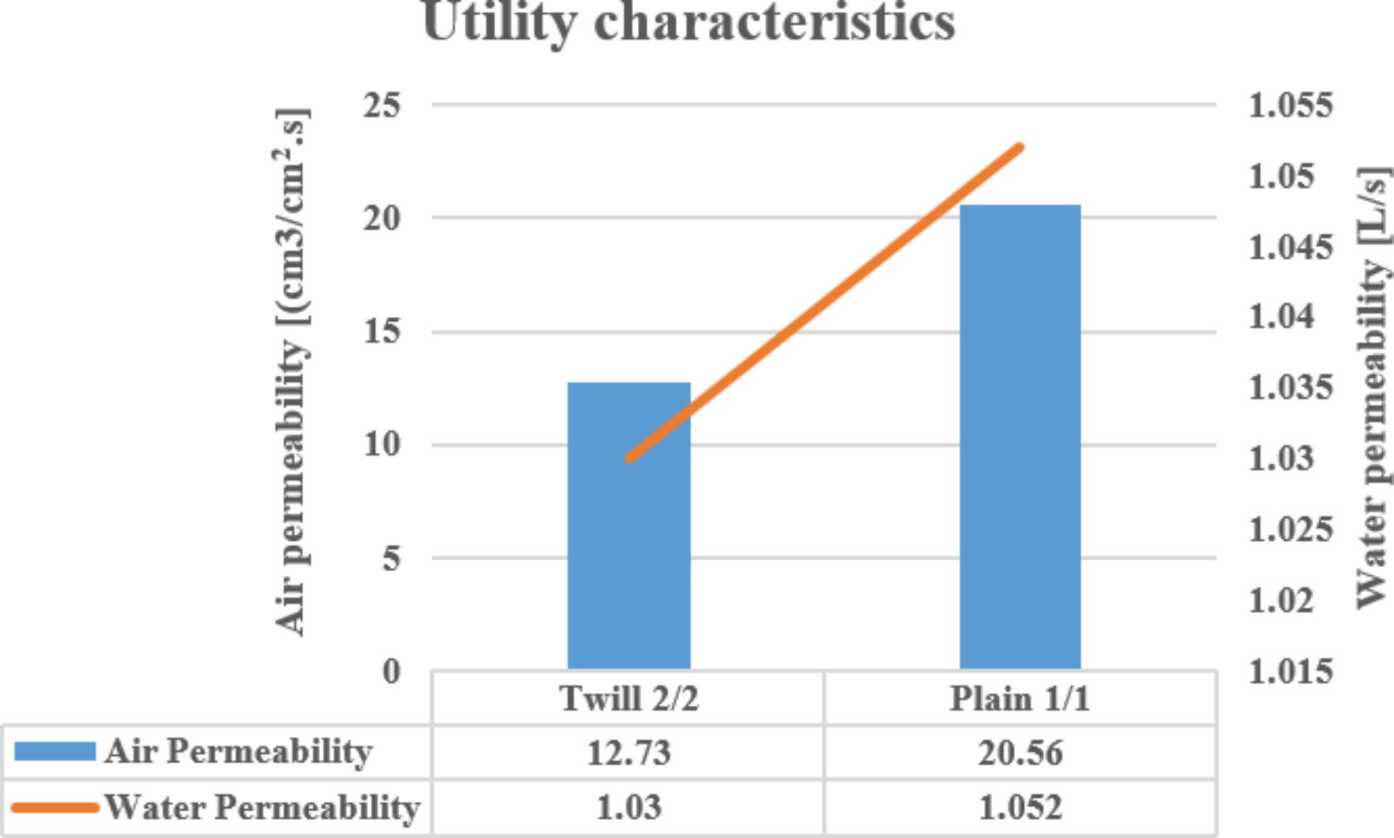
The utility characteristics of the produced woven fabrics.

As shown in Fig. 7 the plain woven structure achieves a higher permeability of air and water compared to the twill woven structure. The justification might have linked to the porosity of the produced fabrics, which correlated positively with the cloth cover factor (fabric cover factor). Whereas decreasing yarn density per unit area reflects on increasing adjacent spacing between interlacing yarns (pore size) which consequently improves air and water flow through the woven fabrics.

### Mechanical characteristics

The results present that produced fabrics have approximately the same tensile strength property either in warp or weft directions. The explanation is traced to the nature of fabric structure and yarn density. The fabric structure reflects on yarn interlacements that consequently affect yarn friction among them and thus on the resistance power of fabric toward mechanical force. Therefore, the higher the yarn interlacements the higher the mechanical force struggle of the woven fabric, due to the natural composition of the utilized woven structures the plain structure has more ability to increase yarn interlacements than the twill structure. Nevertheless, relating to the yarn’s density as displayed in Table 2 twill structure (with more warp and weft yarn density resulting in increased cloth cover factor) generates a mechanical force resistance, which is approximately the same as the plain structure.

**Fig. 8:**
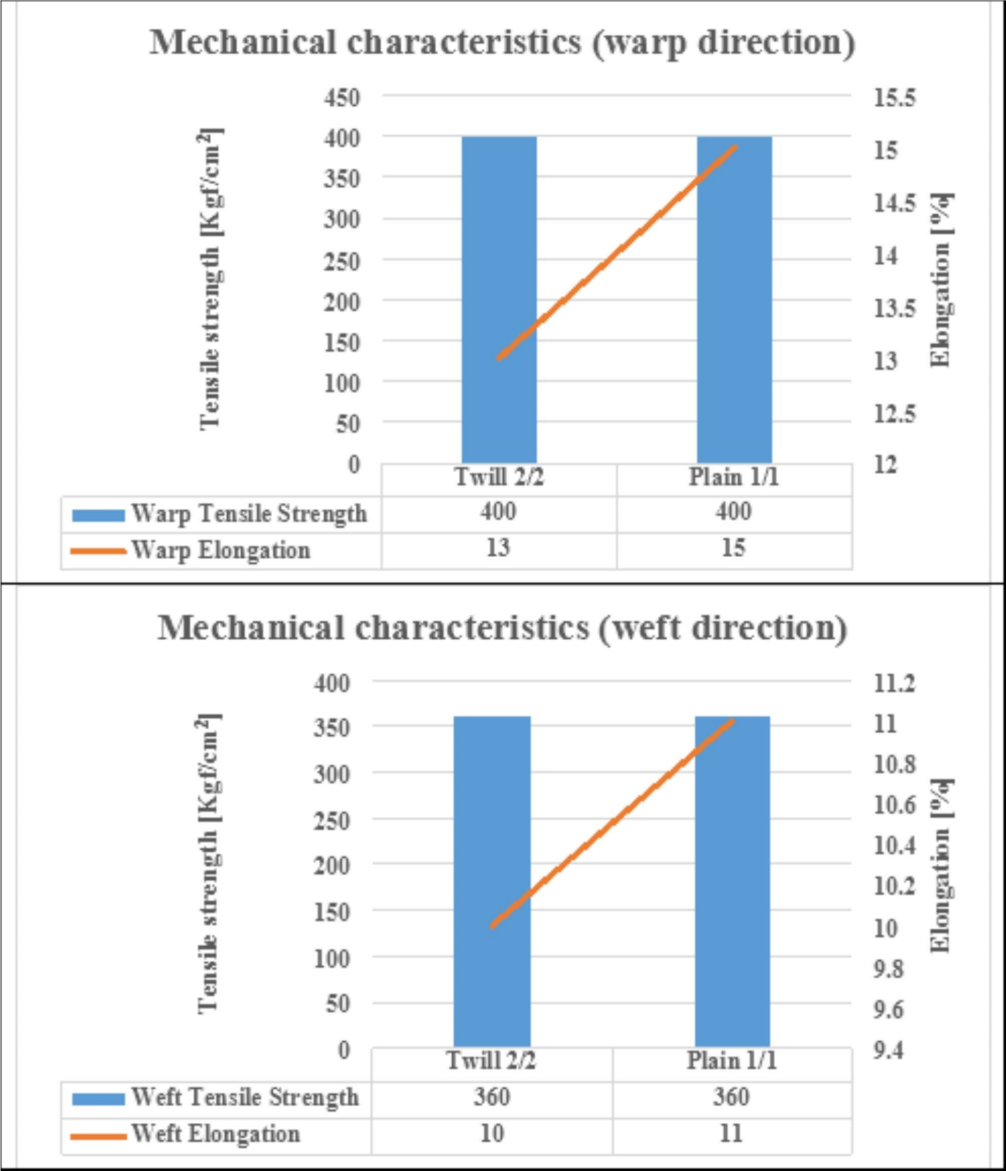
The mechanical characteristics of the produced woven fabrics.

On the other hand, the results refer that woven fabric with plain structure implements more elongation compared to twill structure whether in the warp (vertical) and weft (lateral) directions. The interpretation is linked to the yarn crimp (waviness) that is coupled with yarn intersections, as the higher the yarn crimping the higher the fabric elongation and vice versa. Where the nature of plain structure provides more yarn intersections (more waviness) than twill structure, the fabric extensibility (elongation) will be occurred. Figure 8 illustrates the mechanical characteristics of the produced woven fabrics.

### Significant/insignificant effect

In order to point out the impact of parameters on produced fabrics characteristics, a p-value ≤ 0.05 was statistically determined as revealed in Table 5. The results indicate that thickness and air permeability are significantly affected, presenting that yarn count (diameter) and density play a strike within each fabric structure composition. Moreover, the results specify that areal density, water permeability, and even elongation (warp and weft directions) insignificantly be affected, imparting an enormous effect involving the yarns’ nature (polyester multifilament) at each utilized woven structures more than yarns count and density/cm. Meanwhile, the results designate that there are not any effects for parameters at tensile strength where the produced fabric obtains approximately the same attitude.

**Table 5:** The significant/insignificant effect of parameters.

Sample characteristics	Weave structure		t- statistic	*p*-value	Mean (arithmetic average)
	Twill 2/2	Plain 1/1			
	Mean (arithmetic average)	Differences			
Thickness [mm]	0.46	0.70	0.24	2.833333	0.108^*^
Areal density [g/m^2^]	354	455	101	6.009901	0.052483^**^
Air permeability [(cm^3^/cm^2^.s]	20.56	12.73	7.83	2.251596	0.133041^*^
Water permeability [L/s]	1.052	1.030	0.022	92.63636	0.003436^**^
Warp tensile strength [Kgf/cm^2^]	400	400	0	NA	NA
Weft tensile strength [Kgf/cm^2^]	360	360	0	NA	NA
Warp elongation [%]	13	15	2	12	0.026465^**^
Weft elongation [%]	10	11	1	19	0.016738^**^

(^*^) Insignificant effect, (^**^) Significant effect.

### Crack patterns and mode of failure

**Fig. 9:**
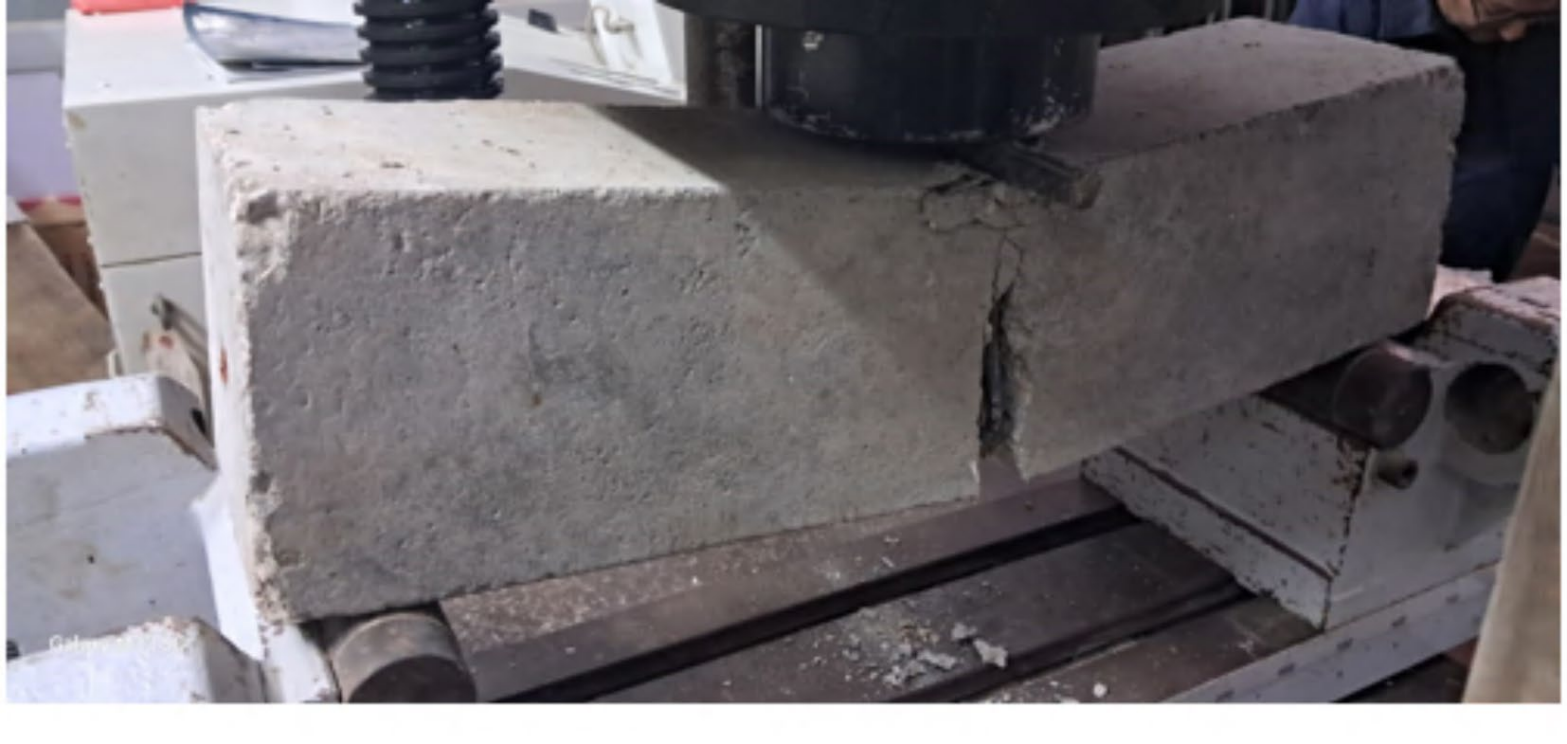
Crack pattern and mode of failure of concrete beam without CNT (B).

**Fig. 10:**
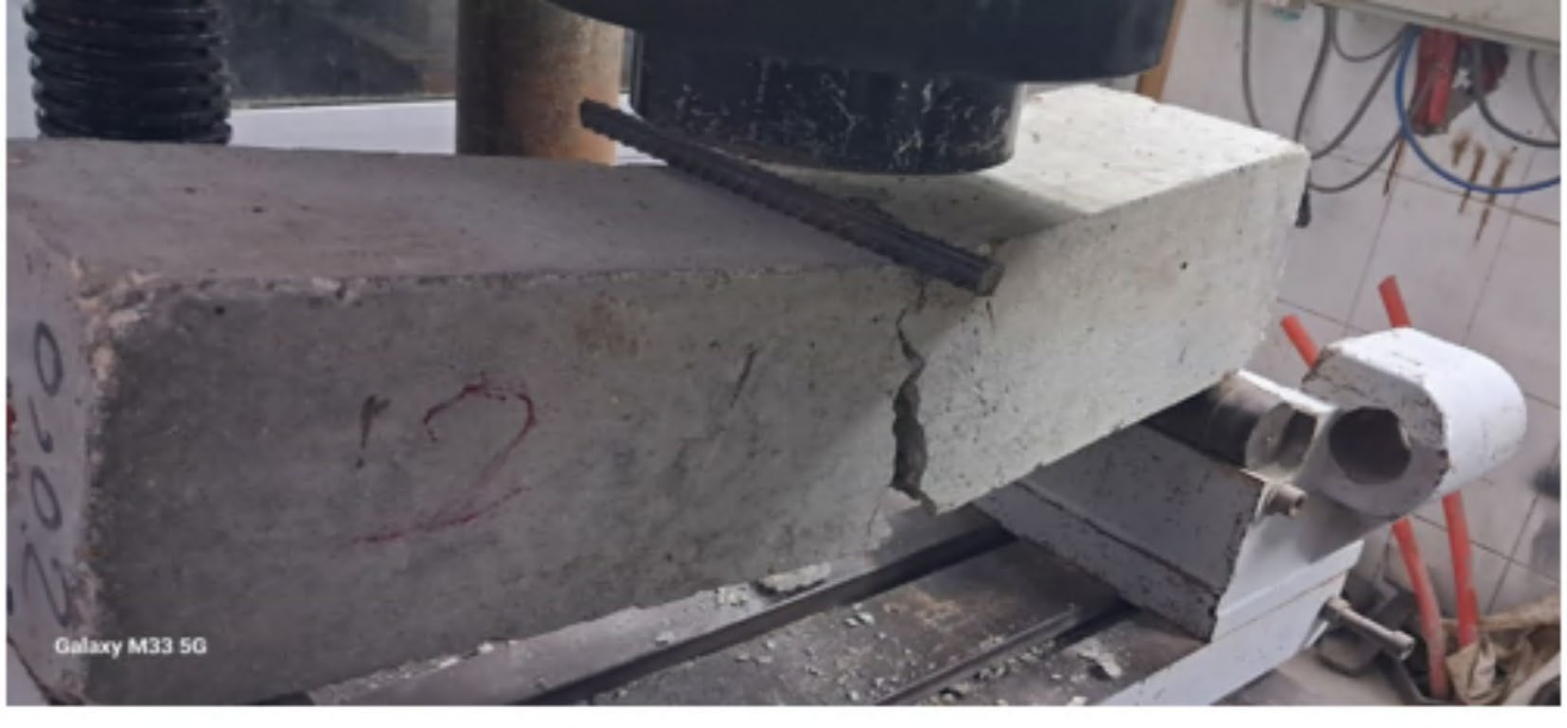
Crack pattern and mode of failure of the concrete beam with 0.02% CNT (BC2).

**Fig. 11:**
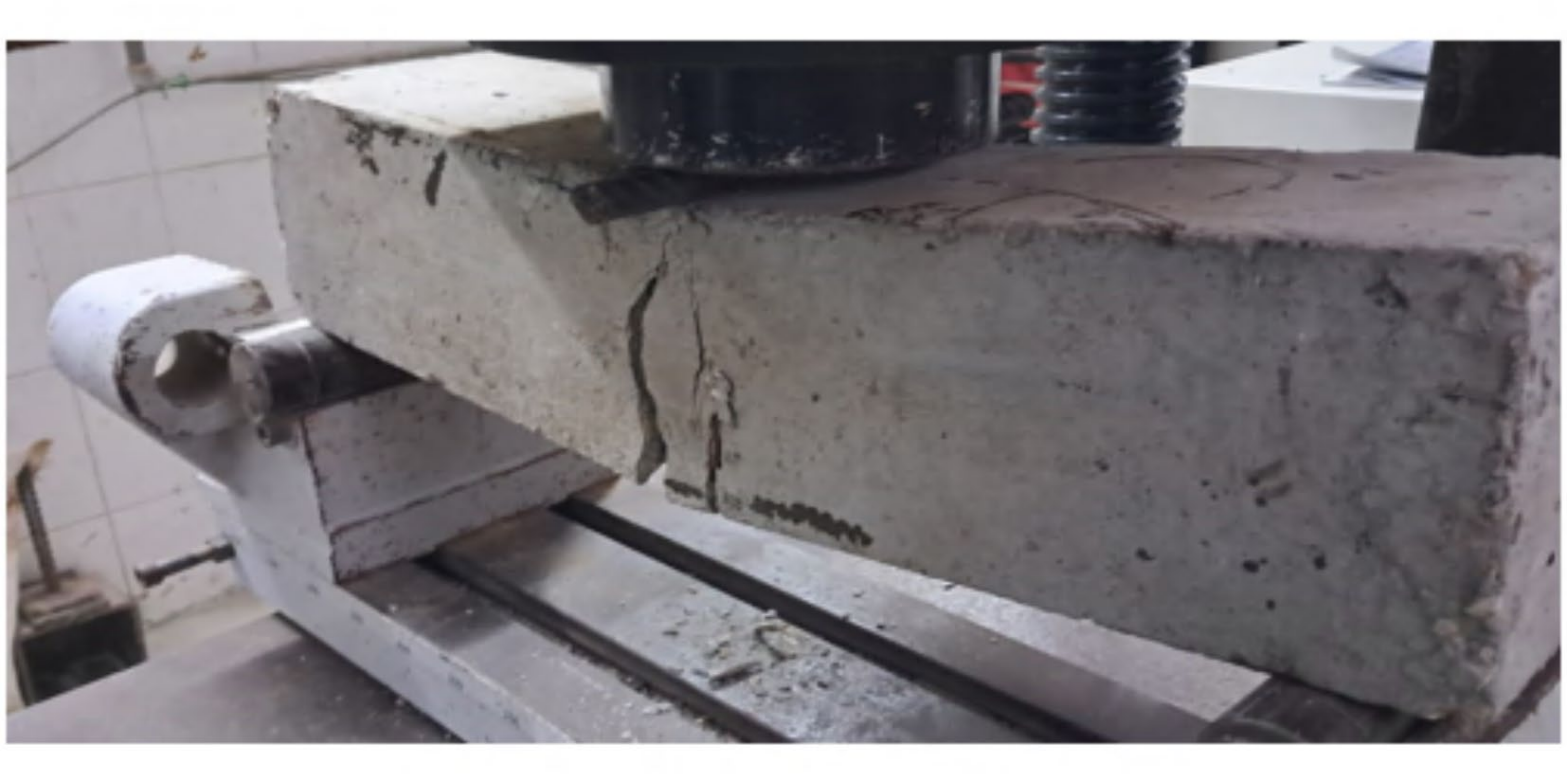
Crack pattern and mode of failure of the concrete beam with 0.04% CNT (BC4).

Figure 9 visually illustrates the flexural cracks observed in beams without the presence of carbon nanotubes (CNTs). These concrete beams exhibited a cracking pattern concentrated at the location of high flexural moment, primarily in or near the middle of the flexural zone. Notably, these beams displayed relatively wide cracks with minimal occurrences of micro cracks compared to the other beams under investigation. Figures 10 and 11 depict the crack patterns observed in concrete beams reinforced with 0.02% and 0.04% CNT, respectively, which also appeared in the middle or near the middle of the flexural zone. These beams exhibited narrower cracks without notable micro cracks, in contrast to the beams without CNT reinforcement. The observed behavior can be attributed to the bridging effect of CNTs, which bridge nano and micro-scale cracks, enhance crack propagation paths within the concrete matrix, and consequently delay crack propagation while increasing the beam’s load-carrying capacity. However, it should be noted that the CNT-reinforced beams displayed relatively wider cracks compared to the beams strengthened with the two textile types.

**Fig. 12:**
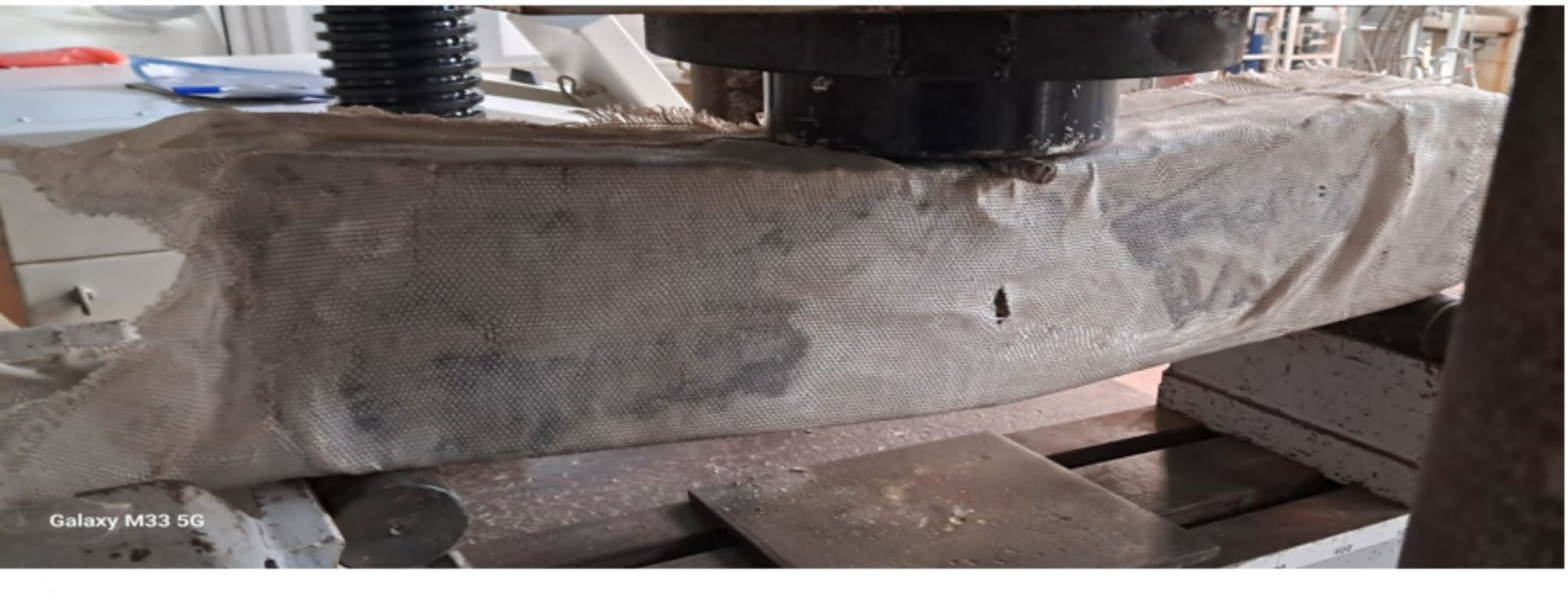
Crack pattern and mode of failure of concrete beam (BS1).

**Fig. 13:**
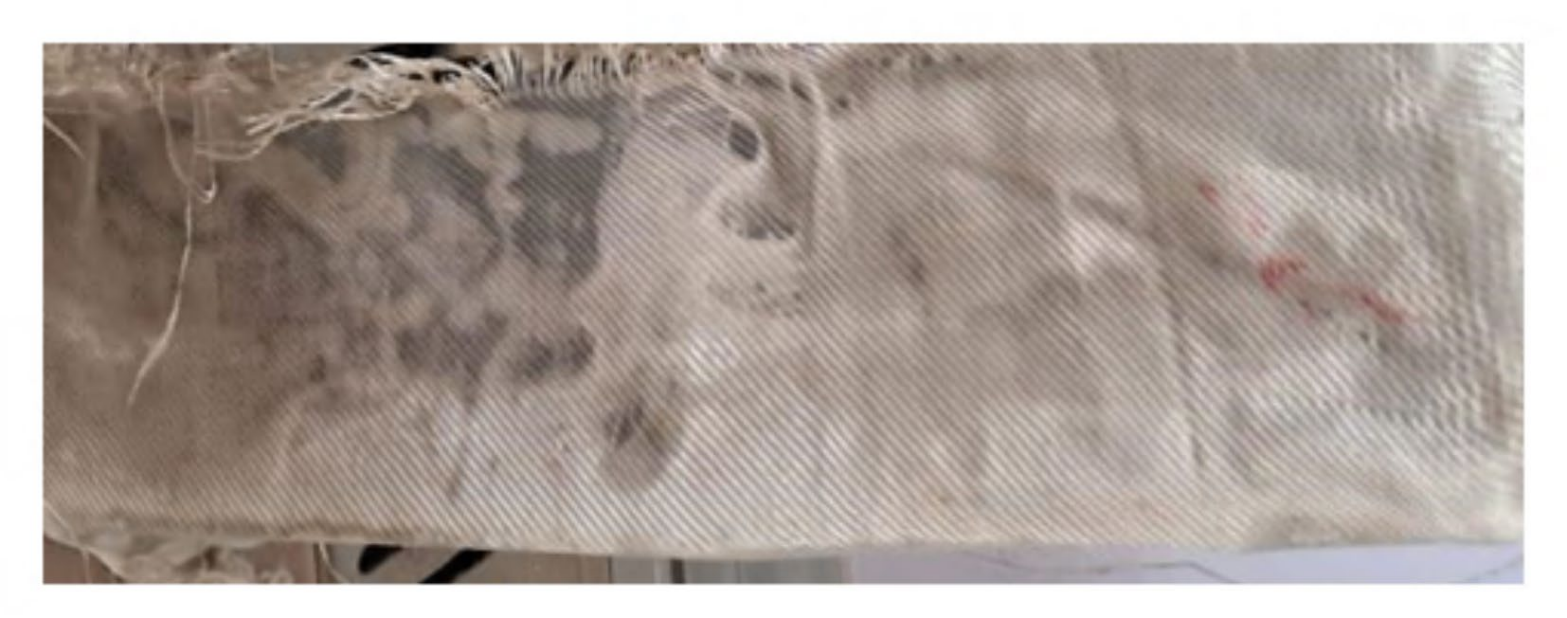
Crack pattern and mode of failure of concrete beam (BS2).

The mode of failure for the concrete beams strengthened with the two textile types exhibited ductile behavior. The failure positions and the initiation of deflections were predominantly located in or near the middle of the flexural zone, as illustrated in Figs. 12 and 13.

Analysis of the aforementioned observations indicates that the inclusion of carbon nanotubes improved the performance of the beams. This improvement can be attributed to the reduction in crack width and the minimized distribution of cracks along the length of the beam, as depicted in Figs. 10 and 11. Regarding the concrete beams strengthened with the two textile types, these beams exhibited ductile behavior and provided clear warning signs before failure due to increased deflection. The failure positions of these beams were concentrated in or near the middle of the flexural zone. This could be attributed to the fact that one of the key characteristics of high-tenacity polyester woven fabrics is the balance between high tensile strength and flexibility, which varies depending on the woven structure composition. This balance allows for easy wrapping around and into various shapes of cement beams. Additionally, high-tenacity polyester yarns possess a degree of moisture absorption that improves the interaction between the bonding material components (epoxy) and the fabric. This enhancement contributes to the overall performance of the cement beams, particularly in reducing the formation of cracks.

### Load-deflection beam behavior

**Fig. 14:**
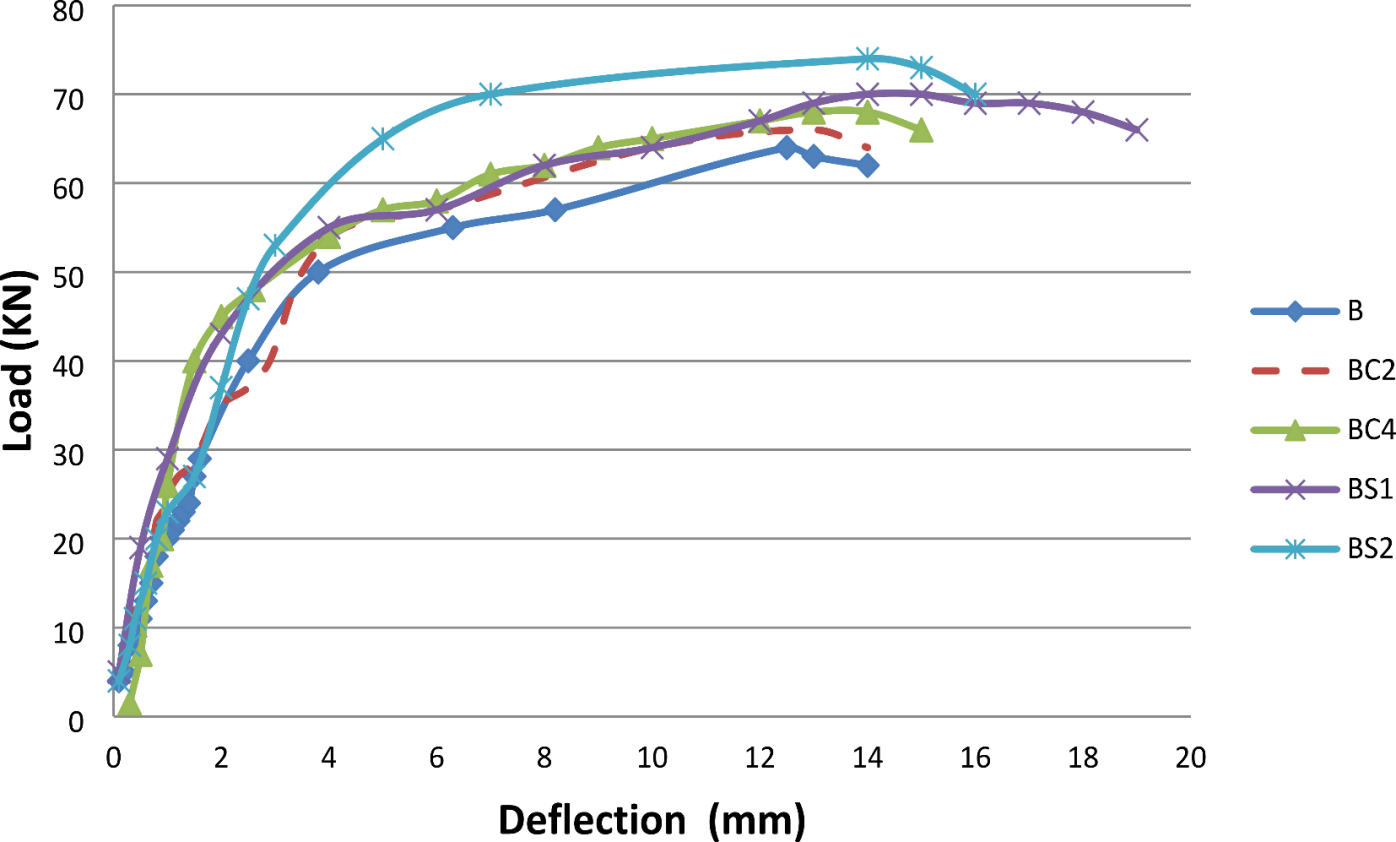
Load - mid span deflection relationship for all beams.

In this study, the behavior of concrete beams under various reinforcement conditions was investigated in relation to load-induced mid-span deflections. The reinforcement conditions included beams without any extra reinforcement (i.e., either CNT’s, or Textiles), beams reinforced with 0.02% and 0.04% carbon nanotubes (CNTs), and textile-strengthened beams with two different textile types (B, BC2, BC4, BS1, and BS2), as depicted in Fig. 14.

The results demonstrated that the textile-strengthened beams exhibited significantly higher post-failure energy absorption capacities under flexure compared to the control beams and the CNT-reinforced beams. This superior performance can be attributed to the generation of multiple cracking patterns in the textile-strengthened beams. The bending response of these beams can be divided into four distinct phases:

- Linearly ascending branch: The external load was primarily borne by the cement matrix until a non-visible crack formed, and the limit of proportionality (LOP) for strength was reached.
- Crack propagation: Multiple cracks formed in this transition zone. The matrix contributed to the composite’s strength in non-cracked zones through tension stiffening, while the reinforcement dominated in the cracked zones. The reinforcement-matrix adhesion maintained the stress transfer mechanism between cracks.
- Post-cracking ascending branch: This branch exhibited a reduced slope due to the degradation of the composite’s stiffness. The fabric reinforcement bridged the cracks and bore the loads, and no new cracks formed in this zone. The cracks mainly widened under the textiles, resulting in significant elongations within the fabrics.
- Failure: Failure occurred due to rupture or debonding of the fabrics, leading to further widening of the cracks under the fabrics and concentration of damage in a single crack.

In contrast, the control and CNT-reinforced beams displayed a relatively brittle response once cracking initiated. Their bending response can be categorized into three distinct branches:

- Elastic range: This branch represents the pre-cracking zone, similar to the textile-strengthened beams.
- Post-cracking regime: This branch exhibits a reduced number of cracks (1–2), resulting in significantly smaller deformation capacity compared to the textile-strengthened beams.
- Pre- and post-failure branches: These branches comprise minimal deflections (less than 2 mm from the onset of cracking), limiting both the ductility and energy absorption capacity. This level of performance may be inadequate for most structural applications in building construction.

Furthermore, the results demonstrate that the properties of the textile samples exhibited a limited influence, except for the angle of wrapping. The addition of CNTs slightly increased the flexural resistance to cracking, with the composite containing 0.04% CNT demonstrating a higher LOP compared to the control and 0.02% CNT beams, attributed to the different distributions of CNT reinforcement within the matrix. The textile-strengthened beams exhibited higher LOP values and greater stiffness compared to the control and CNT-reinforced beams.

In summary, the incorporation of carbon nanotubes enhanced the performance of the concrete beams by reducing crack width and minimizing reasons behind the observed results can be attributed to the bridging effect of the CNTs^5^, which promote crack propagation and delay crack propagation, leading to increased load-carrying capacity. The textile-strengthened beams exhibited superior post-failure energy absorption capacities due to the multiple cracking patterns generated by the fabric reinforcement. This allowed for greater deformation capacity and ductility compared to the control and CNT-reinforced beams. The properties of the textile samples, apart from the angle of wrapping, had a limited influence on the beam behavior. Overall, the study highlights the potential of CNTs and textile reinforcement in improving the performance of concrete beams, providing valuable insights for structural applications in building construction.

## Conclusions

Based on the provided results and discussions, the following can be concluded:

- The plain woven structure achieves a higher areal density and thickness, and promotes air, water permeability and elongation in both warp and weft directions compared to the twill woven structure.
- According to ANOVA test results (with a p-value ≤ 0.05), thickness and air permeability properties were significantly affected by the yarn count (diameter) and density within each fabric structure. Meanwhile, Areal density, water permeability, and elongation (warp and weft directions) properties were not significantly affected, indicating a powerful influence of the high tenacity polyester multifilament yarn nature rather than the count and density/cm.
- The concrete beams lacking carbon nanotubes (CNTs) displayed a cracking pattern concentrated in the middle or near the middle of the flexural zone, characterized by wider cracks and a limited presence of micro-cracks.
- In contrast, the concrete beams reinforced with 0.02% and 0.04% CNTs exhibited narrower cracks and a reduced occurrence of micro-cracks compared to the CNT-free beams. These findings indicate the bridging effect of CNTs, which facilitate crack propagation at both nano- and micro-scales, leading to increased crack paths and delayed crack propagation.
- The failure mode of the concrete beams strengthened with the two types of textile reinforcement demonstrated ductile behavior, with failure points and deflection initiation primarily located in or near the middle of the flexural zone.
- In terms of energy absorption capacity after failure under flexure, the textile-strengthened beams demonstrated significantly higher performance compared to the control beams and those reinforced with CNTs. This superior behavior can be attributed to the generation of multiple cracking patterns facilitated by the textile reinforcement.
- The bending response of the textile-strengthened beams can be categorized into four distinct phases: an initial linearly ascending branch where the external load is primarily borne by the cement matrix until reaching the limit of proportionality (LOP), followed by crack propagation with the formation of multiple cracks. In this phase, the matrix contributes to the strength in non-cracked zones, while the reinforcement dominates in the cracked zones. Subsequently, there is a post-cracking ascending branch with a reduced stiffness, where the fabric reinforcement bridges the cracks and carries the loads. Finally, failure occurs due to the rupture or de-bonding of the fabrics, leading to further widening of the cracks beneath the fabrics.
- Control and CNT-reinforced beams showed a brittle response after cracking, with a limited number of cracks and significantly smaller deformation capacity compared to textile-strengthened beams.
- Textile samples were largely independent of their properties, except for the angle of wrapping.
- The addition of 0.04% CNT increased the crack flexural resistance compared to control and 0.02% CNT beams, due to the different distribution of CNT reinforcement within the matrix.
- Textile-strengthened beams demonstrated higher LOP values and higher stiffness compared to control and CNT-reinforced beams.

## Data Availability

All data generated or analyzed during this study are included in this published article.
